# A Challenging Case of Mechanical Mitral Valve Obstruction

**DOI:** 10.7759/cureus.23945

**Published:** 2022-04-08

**Authors:** Biraj Shrestha, Bidhya Poudel, Tuoyo Mene-Afejuku

**Affiliations:** 1 Internal Medicine, Reading Hospital Tower Health System, Reading, USA; 2 Internal Medicine, AMITA Health Saint Francis Hospital, Evanston, USA; 3 Cardiology, Reading Hospital Tower Health System, Reading, USA

**Keywords:** adult cardiac surgery, mechanical prosthetic valve thrombosis, pannus formation, mitral valve disease, systemic thrombolysis

## Abstract

Prosthetic valve thrombosis (PVT) is a frequent complication with a mechanical valve that presents with symptoms of heart failure or thromboembolic episodes. A 45-year-old lady with antiphospholipid syndrome (APS) complicated by a previous history of native mitral valve thrombus and mechanical mitral valve replacement maintained on warfarin presented with complaints of chest pain and shortness of breath (NYHA class 2). The initial lab showed a subtherapeutic international normalized ratio (INR) of 1.8. Transthoracic echo (TTE) showed severe mitral stenosis with a normal ejection fraction of 65%, elevated peak gradient of 34.5 mmHg, mean gradient of 23.7 mmHg, and pressure half time of 214 ms. Cine-fluoroscopic images revealed an immobile posterior mitral valve leaflet. She failed two trials of low-dose alteplase therapy during the hospitalization. Hence cardiac CT with contrast was done, which showed a small degree of pannus formation on the ventricular surface of the mitral valve ring and a small thrombus. Due to persistent immobility of the post mitral valve after two doses of alteplase and a cardiac CT scan concerning pannus formation, a multi-departmental decision was made to proceed with mechanical mitral valve replacement, following which she had a good recovery. Our case report depicts the importance of imaging study, like cardiac CT scan that can help distinguish thrombus (which has a lower Hounsfield unit, HU of <90) vs. pannus (higher HU of more than 145).

## Introduction

Prosthetic valve thrombosis (PVT) is a life-threatening complication of the mechanical prosthetic valve with a reported incidence of around 0.3%-1.3% patient-years [1]. Potential complications of the mechanical prosthetic valve include thromboembolism, prosthetic mismatch, endocarditis, microangiopathic hemolytic anemia, valve dehiscence, and mechanical mitral valve dysfunction (pannus, dehiscence, valvular, or paravalvular regurgitation) [2]. PVT most commonly presents with progressive dyspnea on exertion, signs of heart failure, or systemic embolization [3]. It is crucial to differentiate PVT from pannus formation as the management differs significantly from surgery vs. trial of thrombolytics [3]. However, at times handling prosthetic valve dysfunction (PVD) can become a difficult task. We present challenges that we faced while managing a case of PVT.

## Case presentation

A 45-year-old female with a history of antiphospholipid syndrome (APS), with mechanical mitral valve replacement, presented to our hospital because of chest pain and shortness of breath on exertion for the last two months. The chest pain was sharp type worse with deep breaths, lasted for minutes, non-radiating, more on exertion, relieved by rest, and associated with shortness of breath. Shortness of breath was also related to exertion and NYHA grade 2. She denied cough, orthopnea, paroxysmal nocturnal dyspnea, fever, sick contacts, palpitation, dizziness, weight gain, or leg swelling. On presentation, blood pressure was 130/80 mmHg, heart rate was 80 beats/min regular, and oxygen saturation was 96% on room air. Physical examination revealed a mid-diastolic murmur in the mitral area and a loud first heart sound. Examination of other systems was unremarkable except for residual right upper limb weakness from a previous stroke which had occurred as a complication of her APS.

She had a past medical history of APS complicated by native mitral valve thrombus resulting in a cardioembolic stroke. She also developed a right middle cerebral artery (MCA) stroke and had a post-stroke seizure due to native mitral valve thrombus embolism. Ultimately, she had to undergo mechanical mitral valve replacement and was started on warfarin. She reported excellent compliance with warfarin. Our initial differentials for shortness of breath and chest pain in this 45-year-old woman were mechanical mitral valve dysfunction (pannus, thrombus, dehiscence, valvular, or paravalvular regurgitation), acute coronary syndrome (ACS), arrhythmia, heart failure, and sub-acute infective endocarditis.

**Figure 1:**
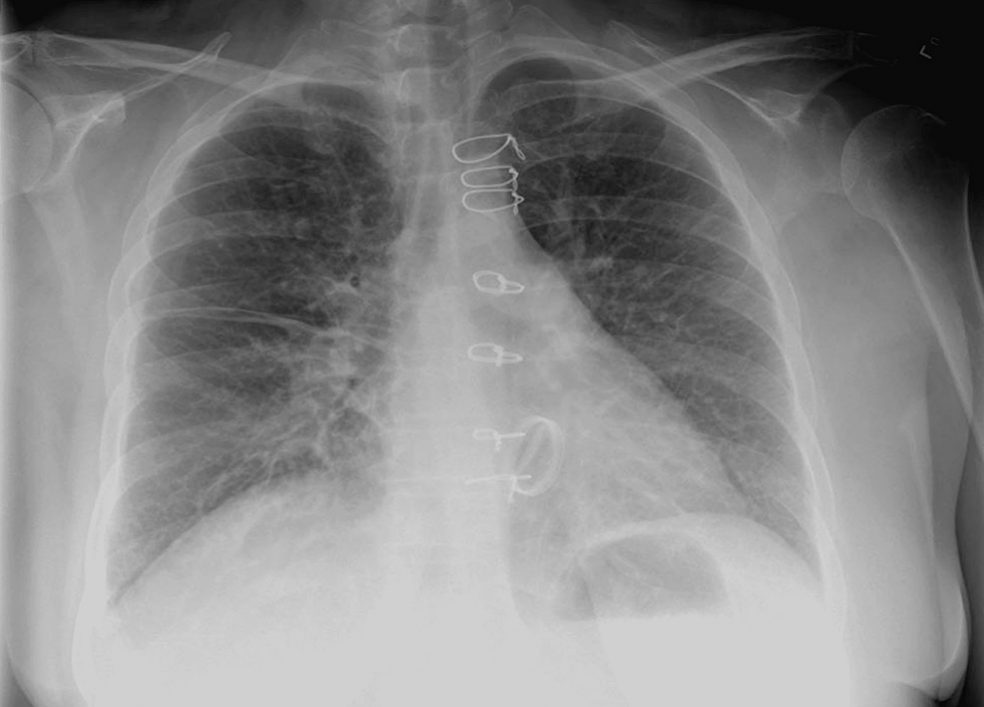
Chest X-ray showing normal cardiac shadow with a mechanical mitral valve in place and a prominent pulmonary vascular marking with bilateral diffuse interstitial edema.

**Figure 2:**
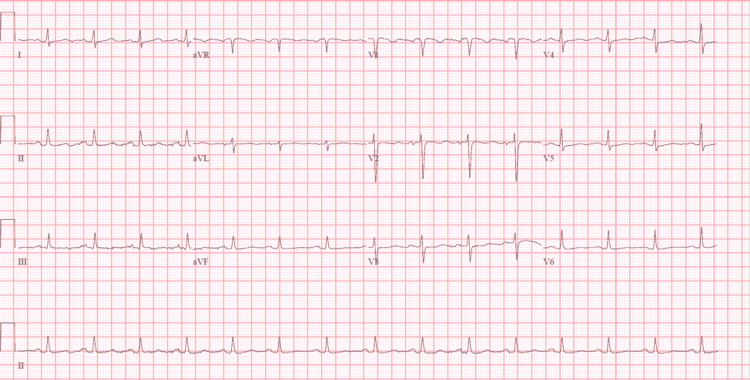
Electrocardiogram showing sinus rhythm 90 beats/min, normal axis, widened P wave (around three small boxes) with M-pattern, normal QRS complex with no significant ST segment or T wave changes.

Her initial lab showed subtherapeutic international normalized ratio (INR) of 1.8 (compared to target INR of 2.5-3.5), troponin I of <0.03 (reference range: < 0.06 ng/mL), and brain natriuretic peptide (BNP) of 408 pg/mL (reference range: 0-100 pg/mL), serum creatine of 0.63 mg/dL (reference range: 0.60-1.30 mg/dL), and hemoglobin of 13.1 g/dL (reference range: 12.0-16.0 g/dL). An electrocardiogram in the emergency department showed sinus rhythm of 90 beats/min, normal axis, widened P wave (around three small boxes) with M-pattern, normal QRS complex with no significant ST segment or T wave changes (Figure 1). Chest X-ray elicited normal cardiac shadow with a mechanical mitral valve and a prominent pulmonary vascular congestion with bilateral diffuse interstitial edema (Figure 2). Transthoracic echo (TTE) showed an ejection fraction of 65%, mechanical mitral valve with a peak transvalvular gradient of 34.5 mmHg, mean gradient of 23.7 mmHg, and pressure half time of 214 ms, and valve area of 1 cm^2^ (based on pressure half time) suggestive of severe valvular stenosis. Further, transesophageal echocardiography (TEE) was done, which showed abnormally functioning bi-leaflet tilting disc mechanical mitral valve prosthesis with a lack of motion of one of the tilting discs and an echo density with a mean forward gradient of 24 mmHg. No prior echocardiograms were found to compare trans-mitral valvular gradients after the mitral valve replacement as she had recently moved from a different state. Fluoroscopy was done, which showed a bi-leaflet mechanical mitral valve with no mobility of one mitral leaflet, and other leaflet was moving freely as seen in Video 1. Blood culture on day three did not show any growth.

**Video 1:** Fluoroscopy showing a bi-leaflet mechanical mitral valve with no mobility of one of the mitral leaflet, and other leaflet was moving freely.

Management

Initially, the patient was continued on warfarin with heparin bridging before TEE was done. On discovering limited leaflet motion concerning thrombus formation and NYHA II heart failure, therapy with tissue plasminogen activator (tPA) was planned. After discussion with the patient, 25 mg of alteplase was administered over 6 h instead of the standard dosing regimen of 100 mg. Following the first dose, the patient reported significant improvement in symptoms, and TTE showed substantial improvement in mean gradient to 10 mmHg. However, repeat fluoroscopy still showed no mobility of one of the mitral leaflets. Hence, the second dose of 25 mg alteplase was administered over 6 h. Unfortunately, repeat TTE still showed a mean gradient across the mitral valve of 9 mmHg, and fluoroscopy failed to show any improvement of the stuck leaflet.

Hence, we were concerned about pannus formation due to the lack of improvement post thrombolytics. Thus, cardiac CT with contrast was done to help identify the etiology of prosthetic valve dysfunction (PVD). As suspected, CT results, based on the Hounsfield unit (HU), were concerning for a small degree of pannus formation on the mitral valve ring's ventricular surface and a small degree of thrombus formation. Due to the lack of further decrease in valve gradient and lack of mobility with consecutive alteplase infusions, a multi-departmental discussion was made to replace the mechanical mitral valve with a 31 mm Saint Jude's mechanical valve. The patient had no perioperative complications, good in-hospital recovery, and was sent to cardiac rehabilitation.

## Discussion

We were suspicious of mechanical mitral valve dysfunction given she was symptomatic with shortness of breath with significant elevation of pressure half lifetime of 214 ms compared to a normal of 25-55 ms (for age 21-72 years) [4], along with TEE and fluoroscopy showing lack of movement of one the valve leaflet. Cine fluoroscopy is the gold standard for detecting mechanical valve dysfunction, but it lacks the capacity for depicting the underlying etiology, just like in our case [5]. The underlying cause of mechanical PVD can be either pannus or thrombus formation, which can be difficult to differentiate [6]. We were initially more inclined toward thrombus formation in our case because of the sub-acute presentation of the patient and initial subtherapeutic INR. Distinguishing points on echocardiography include lower density, irregular shape, attachment to prosthetic heart valve leaflets, or hinge points with mobile mass suggest thrombus formation whereas higher echo density. In contrast, higher density mass extending along the valve ring suggests pannus formation [3]. 

Thrombolytic therapy or surgery remains the primary treatment option for PVT. However, because of a lack of randomized controlled trials and the relative rarity of the condition, the choice between them remains debatable [7]. The current recommendation for surgery includes failure of thrombolytic therapy, sizable thrombus area on TEE of >0.8 cm2, concomitant pannus formation, NYHA class III, IV symptoms on presentation, mobile thrombus, or recurrent valve thrombosis [7]. This was the first episode of mechanical valve thrombosis for our patient with NYHA functional class II symptoms and low clot burden that favored thrombolytic therapy [8]. Therefore, we went for a low-dose slow infusion protocol of 25 mg tPA over 6 h because of the TROIA (comparison of TEE-guided thrombolytic regimen for PVT) trial which compared different treatment strategies that included rapid and slow infusions of streptokinase (groups I and II, respectively), high-dose (100 mg) tPA (group III), one-half dose (50 mg) slow infusion (6 h) of tPA with bolus (group IV), and low-dose slow infusion (25 mg, 6 h) without bolus (group V). Results showed no statistically significant difference in efficacy between the five groups. However, the study noted a lower complication rate (combination of death, major nonfatal complications, and minor nonfatal complications in group V) of 10.5% compared with all other groups with p < 0.05 for each comparison.

After the first dose, the patient's symptoms significantly improved, and TTE showed substantial improvement of the mean gradient to 10 mmHg. However, repeat fluoroscopy still showed no mobility of one mitral leaflet. Hence the second dose of TPA was administered. Unfortunately, despite the success rate of thrombolytic therapy varying from 62% to 81.8% [9-11], TTE post two doses of thrombolysis in our patient still showed a similar mean gradient across the mitral valve, and fluoroscopy still failed to show improvement, which was concerning for pannus formation or a significant clot burden. Hence, to aid with further decision-making regarding management choice, we proceeded with a multi-gated cardiac CT scan to evaluate the lesion's size and extent and distinguish pannus vs. thrombus based on the density seen in the CT scan [12-13]. Pannus has a higher HU of more than 145 with circumferential involvement in contrast to thrombus, which has lower HU (<90) with hinge involvement [14]. Therefore, our case's CT results were concerning for a small degree of pannus formation on the ventricular surface of the mitral valve ring along with a small degree of thrombus formation for which the patient underwent replacement of the mechanical mitral with a 31 mm Saint Jude's mechanical valve. 

## Conclusions

Mechanical PVT can be difficult to manage. Management depends on the size of thrombi, the severity of symptoms, and the presence of pannus. Appropriate imaging studies, like the cardiac CT scan in our case, help distinguish thrombus vs. pannus based on the HU. Therefore, it is of mere importance for the physicians to be acquainted with the above aspects in management for accurate diagnosis and appropriate management.
